# Pharmacokinetics and Bioequivalence Study of Hydroxychloroquine Sulfate Tablets in Chinese Healthy Volunteers by LC–MS/MS

**DOI:** 10.1007/s40744-015-0012-0

**Published:** 2015-07-10

**Authors:** Hong-wei Fan, Zhi-xiang Ma, Jing Chen, Xing-ye Yang, Jun-lin Cheng, Ying-bin Li

**Affiliations:** 1 Department of Clinical Pharmacology Laboratory, Nanjing First Hospital, Nanjing Medical University, 210006 Nanjing, Jiang Su China; 2 XenoBiotic Laboratories China, 210038 Nanjing, China; 3 The 2nd Affliated Hospital of Nanjing Medical University, 210011 Nanjing, China

**Keywords:** Bioequivalence, Hydroxychloroquine (HCQ), Pharmacokinetics

## Abstract

**Introduction:**

Hydroxychloroquine (HCQ), 4-aminoquinoline, is an antimalarial drug and has become a basic therapy for rheumatic disease treatment. It can stabilize the condition of SLE patients and reduce the chances of patient relapse through its immunosuppressive function and antiinflammatory effects. This drug was absorbed completely and rapidly by oral administration, but has a prolonged half-life for elimination. The objective of this study was to evaluate the pharmacokinetic parameters and relative bioequivalence of a new generic (test) formulation with the branded (reference) formulation of HCQ in healthy Chinese male volunteers. This study was designed to acquire regulatory approval for the test formulation.

**Methods:**

This study was conducted with a randomized, single-dose, two-period, and crossover design. The male subjects were randomly assigned to two groups at a 1:1 ratio to receive 0.2 g hydroxychloroquine sulfate tablets (0.1 g/piece) of the two formulations after a 3-month washout period then administered the alternate formulation. Study drugs were administered after overnight fasting (over 10 h). Plasma concentrations of hydroxychloroquine were measured by a validated LC-MS/MS method. The following pharmacokinetic properties were determined by a noncompartmental pharmacokinetic method: *C*_max_, *T*_max_, AUC_0–*t*_, AUC_0–∝_, and *t*_1/2_. The bioequivalence between the test and reference products was assessed based on the following parameters: *C*_max_, AUC0–60d, and AUC_0–∝_ using the ANOVA method. If the 90% CI for AUC_0–*t*_ was within 80–125% and for *C*_max_ was within 70–143% of the statistical interval proposed by the SFDA, the two formulations were assumed bioequivalent. Concerning the main pharmacokinetic charateristics of hydroxychloroquine, a long half-life drug, the pharmacokinetic parameters of 0–72 h were determined according to the FDA. Furthermore, a comparison was made between the parameters at 0–60 days and 0–72 h to evaluate whether a truncated AUC method can be applied to estimate the relative bioavailability of HCQ. Tolerability was assessed by monitoring vital signs and laboratory tests and by questioning subjects about adverse events.

**Results:**

The 90% CI of *C*_max_ for HCQ is 103.8–142.3%; the AUC0–60 is 100–114.2% and AUC_0–∝_ 100–115.5%. Both met the criteria according to the SFDA’s guidelines for bioequivalence. The relative bioavailability was 109.5% (according to AUC_0–60d_) and 110.7% (according to AUC_0–∝_). No serious or unexpected adverse events were observed.

**Conclusions:**

In this study, the pharmacokinetic studies and results were conducted so that the test and reference formulations of HCQ met the Chinese criteria for assuming bioequivalence. Both formulations were well tolerated in the population studies.

## Introduction

Hydroxychloroquine (HCQ) is a 4-aminoquinoline that differs from chloroquine (CQ) by the addition of a hydroxyl group. It was first synthesized in 1944 and initially used as an antimalarial agent [1]. The activity of HCQ against malaria is equivalent to that of CQ, and HCQ is preferred over CQ when high doses are required because of its lower level of ocular toxicity [2]. Both drugs have now become mainstays in the management of rheumatic diseases: principally systemic lupus erythematosus (SLE) and rheumatoid arthritis (RA) [3, 4]. Now HCQ is considered the second-line treatment for SLE, but it is quite effective therapeutically. One study showed that HCQ can reduce the risk of clinical SLE flares and severe SLE exacerbations [5]. Insulin resistance occurs more frequently in RA and SLE and is a common risk factor for cardiovascular disease (CVD) and diabetes mellitus (DM) [6–8]. A study showed that HCQ has a beneficial effect on insulin sensitization by using HCQ in non-diabetic obese subjects for 6 weeks [9]. HCQ also appears to protect against the occurrence of thrombotic events [10]. In addition, the main mechanism of HCQ applied in rheumatic diseases is the inhibition of stimulation of Toll-like receptors (TLRs). TLRs are cellular receptors for microbial products that induce inflammatory responses by activating the innate immune system [11]. Two types of side effects may be encountered with HCQ treatment: one is gastrointestinal intolerance, which usually disappears with dose reduction; the other is rare but potentially severe and involves various combinations of retinal, neuromuscular, cardiac, and hematological impairments. Compared with CQ treatment, retinopathy’s incidence in HCQ treatment is very small [12].

HCQ is rapidly and almost completely absorbed after oral administration (the absorption rate through the gastrointestinal tract is 70–80%). However, it has a prolonged half-life (between 40 and 50 days) and low blood clearance (96 ml/min) because of its PK properties [13]. Approximately 50% of the HCQ in plasma is bound to plasma proteins. In the liver, HCQ is metabolized to three active metabolites: desethyl-chloroquine (DCQ), desethyl-hydroxychloroquine (DHCQ), and bis-desethyl-hydroxychloroquine (BDCQ) [14].

Although the pharmacokinetic properties of HCQ have been well identified in previous studies, few studies have been conducted on the PK characteristics of HCQ among healthy Chinese individuals. Although one similar study reported on the bioequivalence of HCQ, it had a parallel study design. However, according to the findings of Tett et al. [16], the bioavailability of HCQ is consistent in each individual at different times but variable between subjects. The FDA also advises that a crossover study design can make the variables determined by physiological factors (such as the clearance, volume of distribution, and absorption) have less interoccasion variability than that arising from formulation performance. Therefore, differences between two products because of formulation factors can be determined [17]. Thus, this study was conducted using a crossover design. Compared with the preceding study design, this one could eliminate individual differences. Before a generic product can be marketed in China, the State Food and Drug Administration (SFDA) requires a bioequivalence experiment. The aim of this study was to compare the relative bioavailability of a new generic formulation (test) of HCQ and the branded formulation (reference) in a Chinese population to meet the SFDA’s requirement for marketing the generic formulation in China.

## Methods

### Materials

Hydroxychloroquine sulfate was purchased from C & O Pharmaceutical Technology (Holdings), Ltd., (Nanjing, China), lot no. 090526. Chloroquine phosphate was purchased from The National Institute for the Control of Pharmaceutical and Biological Products, lot no. 100421-200401. The reference formulation was obtained from Shanghai Zhongxi Pharmaceutical Co., Ltd., Shanghai, China, lot no. 081203. The test formulation was obtained from Jiangsu Shenhua Pharmaceutical Co., Ltd., Jiangsu, China, lot no. 20090721. HPLC grade methanol, acetonitrile, and formic acid were all purchased from Merck. Milli-Q deionized water was used throughout the study.

### Instruments

For analysis, a Shimadzu UFLC-20 AD XR liquid system interfaced to an Applied Biosystems API-5000 tandem quadrupole mass spectrometer (analysis software Analyst 1.5) was equipped with a 50 × 2.1-mm 2.6-μm C18 column (Kinetex, Phenomenex) at room temperature.

### Study Design and Procedures

Healthy Chinese males aged 18–40 years with BMIs between 19 and 24 kg/m^2^ were enrolled in the study. Subjects were considered healthy on the basis of medical history, full physical examination, clinical laboratory tests (especially for renal and hepatic function), vital signs (oral body temperature, heart rate, respiratory rate, and sitting blood press), and 12-lead ECGs. Subjects were excluded if they had any impairment of a major organ; had used or abused an illegal drug or alcohol; had psoriasis, active bleeding, colds, or clinically significant abnormalities or hydroxychloroquine-like eye lesions after inspection of the fundus; had a history of mental or neurological disease or glucose-6-phosphate dehydrogenase (G-6-PD) defects; had an allergy or sensitivity to 4-aminoquinoline compounds; or had participated in a clinical trial within 2 weeks or donated blood within 2 months prior to the study. The subjects had been informed about the details, including the risks and benefits of this study, and they were free to withdraw at any time.

The study was conducted according to a randomized, open-label, single-dose, two-period, and crossover design. Subjects were assigned randomly to two groups to receive a single dose of 0.2 g hydroxychloroquine sulfate tablets (0.1 g/piece) with 250 ml water in the test formulation (Jiangsu Shenhua Pharmaceutical Co., Ltd., Jiangsu, China; lot no. 20090721) or the reference formulation (Shanghai Zhongxi Pharmaceutical Co., Ltd., Shanghai, China; lot no. 081203). Subjects were required to take tablets after overnight fasting (over 10 h) and without breakfast, and they were not allowed to drink alcohol, coffee, and juice, but were allowed to drink water 2 h after administration. They were allowed to have a standard meal 4 h after administration. There was a 3-month drug-free washout period followed by administration of the initial formulation after the alternate formulation had been administered. At the end of the test, subjects were scheduled to have re-examinations of routine blood tests and alanine aminotransferase, aspartate aminotransferase, and creatinine levels.

Blood samples (3 ml each) were collected from a suitable forearm vein by an indwelling catheter at the following time points: before dosing (baseline), 1, 2, 3, 4, 5, 7, 9, 12, 24, 48 h (day 3), 72 h (day 4), 120 h (day 6), day 10, day 20, day 40, and day 60 after administration. Blood samples were collected into tubes containing sodium-heparin as anticoagulant then immediately stored in a container filled with ice. Samples were handled within 30 min after collection. Plasma was stored frozen (−65°C) in labeled polypropylene tubes until analysis.

### Drug Assay

#### Stock and Working Solutions

Stock solutions of hydroxychloroquine and chloroquine (Inner Standard, The National Institute for the Control of Pharmaceutical and Biological Products, Beijing, China; Lot no.100421-200401) were prepared in water (1 mg/ml) and further individually diluted with water to obtain the desired concentrations: hydroxychloroquine, 100 µg/ml; chloroquine (IS), 10.0 µg/ml and 100 ng/ml. The stock solutions were kept refrigerated and restored to room temperature before use.

#### Calibration Standards and QC Samples

The standard curve ranged from 0.200 to 100 ng/ml (*r* = 0.9930). Eight calibration standards were prepared according to the preparation of stock solutions; their concentrations were 0.200, 0.400, 2.00, 5.00, 10.0, 20.0, 50.0, and 100 ng/ml. There were double samples at one concentration. The quality control samples were prepared at concentrations of 0.500 (low), 15.0 (medium), and 80.0 ng/ml (high); six samples were prepared at each concentration, as a single batch at each concentration.

#### Sample Preparation

All frozen human plasma samples were first restored to room temperature. A 50-μl volume of plasma was transferred to a micro-centrifuge tube (1.5 ml); 0.3 ml acetonitrile was added sequentially and vortex-mixed for 3 min. After centrifugation at 4000 rpm (4°C) for 10 min, 150 μl supernatant was pipetted and dried by nitrogen (35°C), then reconstituted with 200 μl 20% methanol solution, vortexed, and centrifuged at 4000 rpm for 5 min. The supernatant was pipetted for assay.

#### Chromatographic Conditions

The samples were injected onto a 50 × 2.1-mm 2.6-μm C18 column (Kinetex, Phenomenex) at room temperature. The mobile phase comprised 0.6% formic acid aqueous solution (phase A) and methyl alcohol (80:20, v/v) (phase B) at a flow rate of 0.5 ml/min. The column temperature was at room temperature.

#### Mass Spectrometric Conditions

The analytes were quantified by mass spectral detection using a mass spectrometer (API 5000, AB SCIEX) equipped with an Turbo-ESI source, which runs in positive mode. The mass spectrometer was set up in MRM mode to monitor the transitions 336.2 → 247.2 and 320.2 → 247.2 for hydroxychloroquine sulfate and IS, respectively. The capillary voltage was set at 1.5 kV. The desolvation temperature was 550.

### Validation

#### Linearity

Eight calibration standards were prepared according with the preparation of stock solutions; their concentrations were 0.200, 0.400, 2.00, 5.00, 10.0, 20.0, 50.0, and 100 ng/ml. There were double samples at one concentration. The standard curves were calculated by the equation *f* = *aC* + *b* using weighted (1/*C* ^2^) least square regression.

#### Lower Limit of Quantitation

**Fig. 1:**
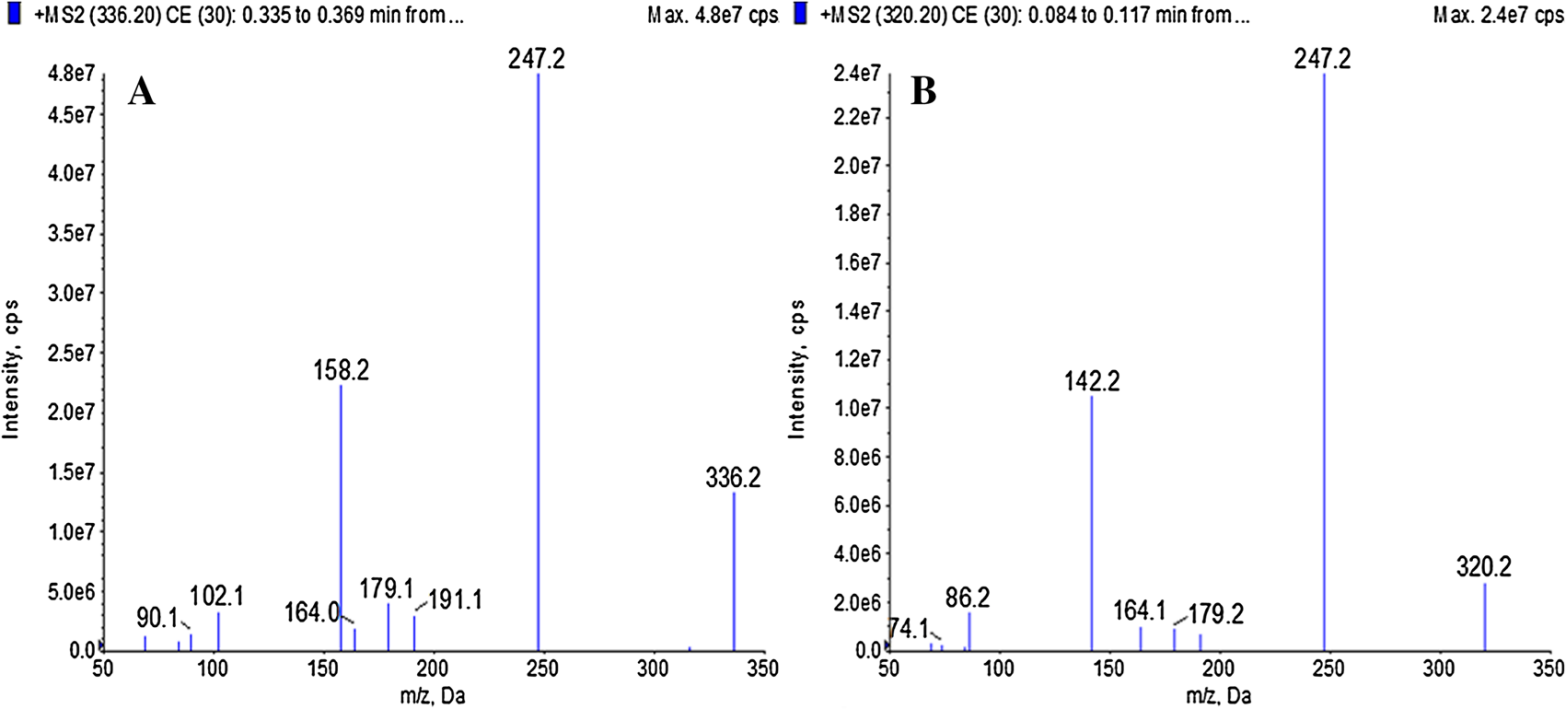
Full-scan product ion spectra of **a** hydroxychloroquine sulfate and **b** chloroquine phosphate

Six copies of plasma containing standard hydroxychloroquine at a concentration of 0.200 ng/ml were prepared and analyzed by LC-MS/MS. According to the chromatograms, the calculated *f* (the ratio of peak areas between the hydroxychloroquine solution and internal standard) was then substituted into the standard curve obtained on that day to determine the actual concentration. Eventually, we calculated the accuracy and RSD. The results suggested that the lower limit of quantitation could meet the acceptance criteria (Fig. 1).

#### Specificity

**Fig. 2:**
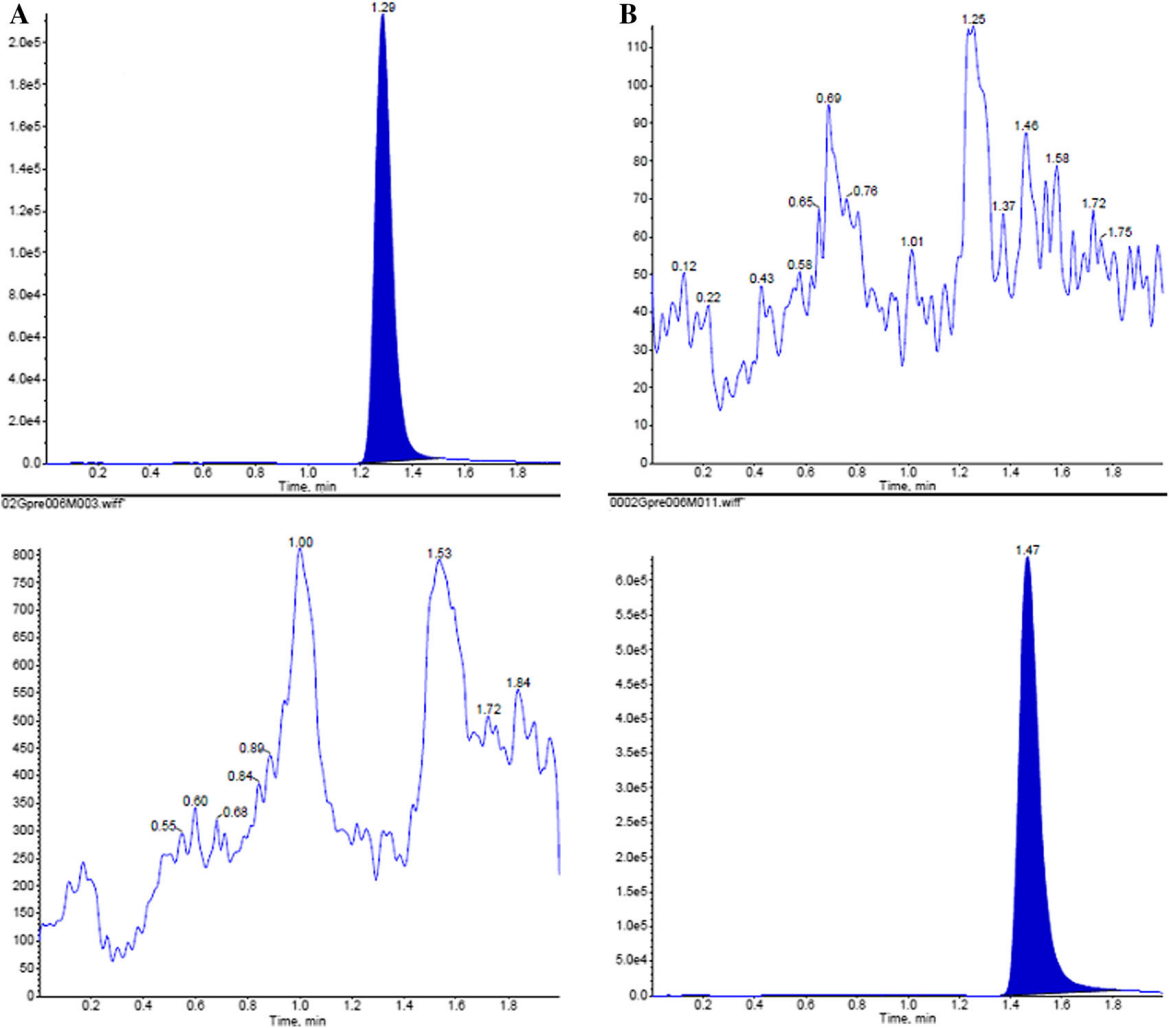

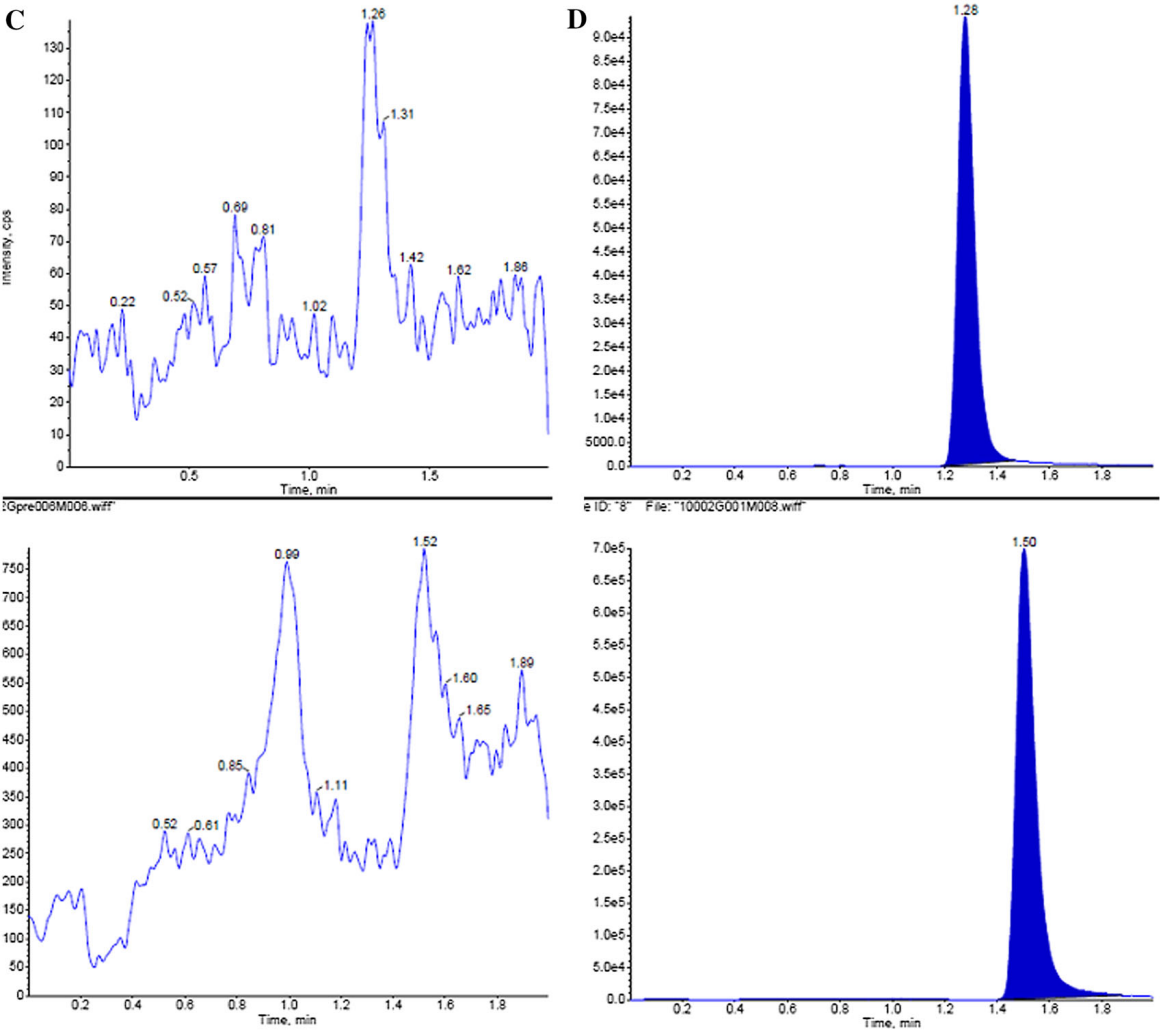

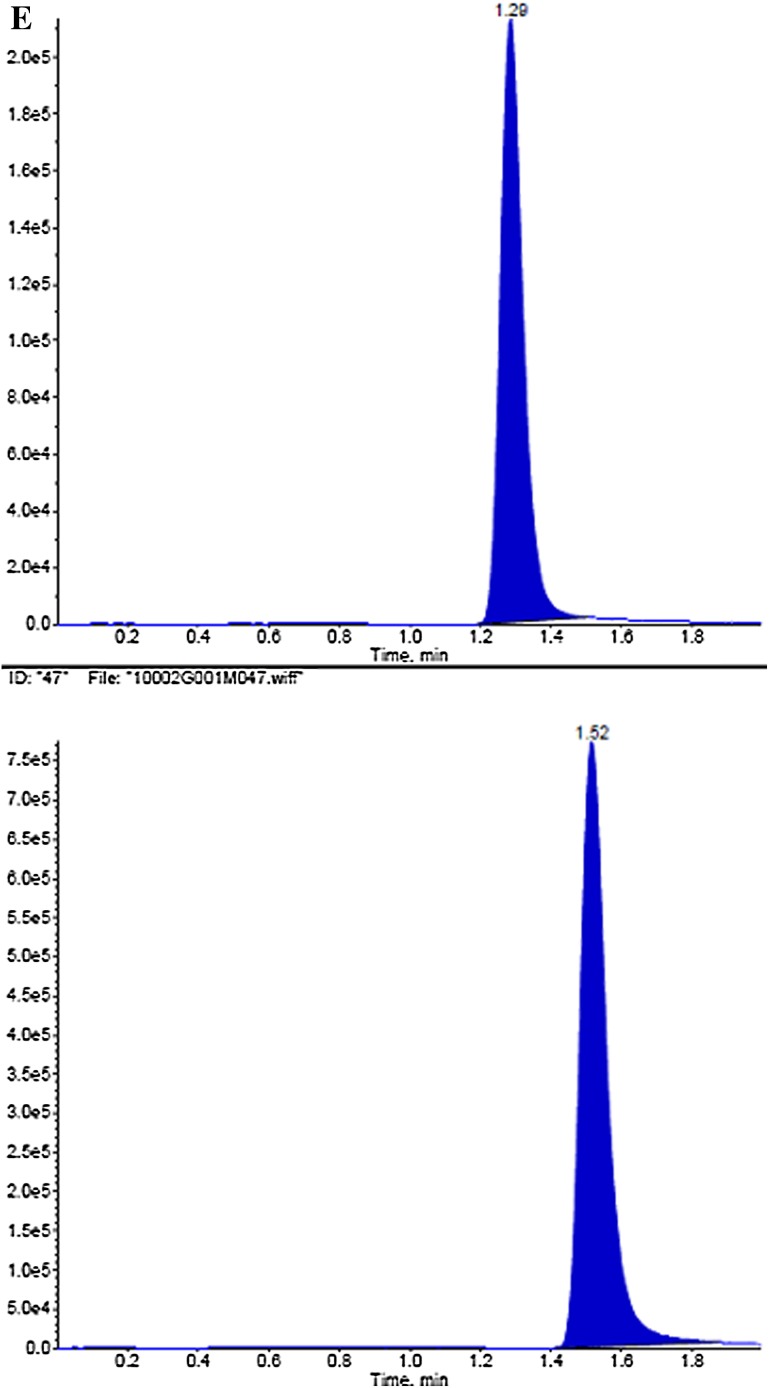
**a** Hydroxychloroquine reference chromatogram; **b** internal standard reference chromatogram; **c** blank plasma chromatogram; **d** blank plasma plus hydroxychloroquine and internal standard reference chromatogram; **e** plasma samples collected from healthy subjects taking hydroxychloroquine sulfate tablets orally 200 mg after 1 h chromatogram. Hydroxychloroquine (*upper*): *m/z* 336.2 → 247.2; internal standard (*lower*): *m/z* 320.2 → 247.2

Six randomly selected control blank human plasma samples were processed by a similar extraction procedure and analyzed to determine whether the hydroxychloroquine and chloroquine peaks were well shaped and no impurity peaks interfered with the determination under the chromatographic conditions used in this study. The results in Fig. 2 suggest that the conditions provided high specificity and sensitivity (Table 1) and can accurately determine the concentration of plasma hydroxychloroquine.

**Table 1:** RSD of lower limit of quantitation of hydroxychloroquine plasma samples

Concentration (ng/ml)	Measured concentration(ng/ml)	Accuracy (%)	Mean of measured concentration (ng/ml)	RSD (%)
0.200	0.195	97.50	0.185	5.20
	0.179	89.50		
	0.191	95.50		
	0.173	86.50		
	0.193	96.50		
	0.176	88.00		

#### Recovery and Matrix Effect

The recovery was evaluated by the response of the analyte recycled from the biological sample matrix divided by the response of the pure standard. The matrix effect experiments were performed by evaluating the ratio between the spiked mobile phase solutions and un-extracted samples spiked on plasma residues (Table 2).

**Table 2:** Extraction recovery and method recovery of HCQ assays in plasma (*n* = 3)

Concentration (μg l^−1^)	Extraction recovery (%)	Method recovery (%)
0.50	98.56 ± 6.87	91.87 ± 5.59
15.0	97.66 ± 5.43	98.22 ± 3.29
80.0	95.64 ± 5.77	97.84 ± 5.51

#### Accuracy and Precision

Both the accuracy and precision evaluations were performed by repeated analysis of hydroxychloroquine in human plasma. The run consisted of a calibration curve and six replicates each of LLOQ and the low-, medium-, and high-quality control samples. Three batches were measured respectively, recording the chromatograms and then calculating *f* (the ratio of peak areas between the hydroxychloroquine solution and internal standard), obtaining the accuracy of the measured concentrations through the standard curve. Eventually, we calculated the accuracy values between and within batches. The acceptance criterion for each batch was that the deviation of the calculated concentrations should be within ±15.0% of their theoretical concentrations (±20.0% for lower limit of quantification). The results shown in Table 3 indicate that the assay method is reproducible for replicate analysis of hydroxychloroquine in human plasma.

**Table 3:** Intra-group and inter-group precision of HCQ assays in plasma (*n* = 6)

Concentration (μg l^−1^)	Intra-group	Inter-group
0.50	3.04	9.05
15.0	4.02	8.13
80.0	5.63	7.26

#### Stability

The stability of hydroxychloroquine in plasma was evaluated in the following studies: a stability study at room temperature, a stability study in an auto-sampler, and a freeze-thaw study. LLOQ and QC samples (0.500, 80.0 ng ml^−1^) of hydroxychloroquine were assayed among three batches.

#### Pharmacokinetic Analysis

Pharmacokinetic properties were determined by a noncompartmental pharmacokinetic method. The following parameters were calculated for each subject during each session: *C*_max_, *T*_max_, AUC_0–*t*_, AUC_0−∝_, and *t*_1/2_. *C*_max_ and *T*_max_ were obtained directly from the plasma concentration versus time curve. Other parameters (AUC_0–*t*_, AUC_0–∝_ and *t*_1/2_) were calculated by noncompartmental analysis using DAS version 2.0 (Shareware Software, Shanghai, China). AUC_0–*t*_ was calculated using the trapezoidal method and AUC_0–∝_ as AUC_0–*t*_ + Ct/ke, where ke is the terminal elimination rate constant calculated by linear least squares regression analysis using the points in the terminal log-linear phase from the plasma concentration versus time curve. *t*_1/2_ represents the apparent first-order terminal elimination half-life, calculated as 0.693/ke. The relative bioavailability (F) of the tested formulation was calculated as AUC_0–*t*_ (test)/AUC_0–*t*_ (reference) × 100%.

The bioequivalence between the test and reference products was assessed based on the following parameters: *C*_max_, AUC0–60d, and AUC_0−∝_. An ANOVA using DAS version 2.0 for a double crossover experimental design was used to compare the log-transformed C_max_, AUC0–60d, and AUC_0–∝_ values. Period, subject, and sequence effects were determined at a significance level of *α* = 0.05. The 90% CIs served as interval estimates and were determined using two one-sided *t* tests. If the differences in PK parameters between the two formulations were not statistically significant (*P* > 0.05) and the 90% CI for AUC_0–*t*_ was within 80–125% and for *C*_max_ was within 70–143% of the statistical interval proposed by the SFDA, then the two formulations were considered to have met the regulatory requirements for bioequivalence.

### Tolerability

Throughout the study, subjects were monitored by two doctors, two pharmacists, and two nurses. Tolerability was assessed based on vital signs (blood pressure, heart rate, breathing rate), clinical laboratory tests, and 12-lead ECGs. Physical examinations were performed at baseline and after completion of the study; subjects were interviewed about symptoms of possible adverse events (AEs). Once any undesirable symptoms occurred in subjects, the information would be recorded on the CRF, and the subjects would countinue to be monitored until their physical condition returned to normal.

## Results

### Study Population

Twenty-one healthy Chinese male volunteers participated in this study; 20 volunteers eventually completed the study. The patient characteristics [mean (SD)] were as follows: age, weight, height, and body mass index respectively were 22 [2.9] years, range 21 to 29 years; 69 [6.2] kg, range 54 to 75 kg; 174 [5.1] cm, range 166 to 183 cm; 23 [1.3] kg m^−2^, range 19 to 24 kg m^−2^.

### LC/MS/MS Method Validation

The linear range of hydroxychloroquine was 0.20–100 ng/ml (*r* = 0.9911). Moreover, the accuracy of the range from 0.20 to 100 ng/ml was between 87.60 and 104.13%, the inter-analysis RSD was <6.0%, and the intra-analysis RSD was <6.2%. For LLOQ, the accuracy ranged from 88.00 to 97.50%. The matrix effect and recovery [mean (SD)] of hydroxychloroquine were 102.40 [2.18] and 98.23 [2.28]%; for IS they were 99.08 [1.62] and 93.16 [5.87]%, respectively.

### Statistical Evaluation of Pharmacokinetic Parameters

#### 0–60d

**Fig. 3:**
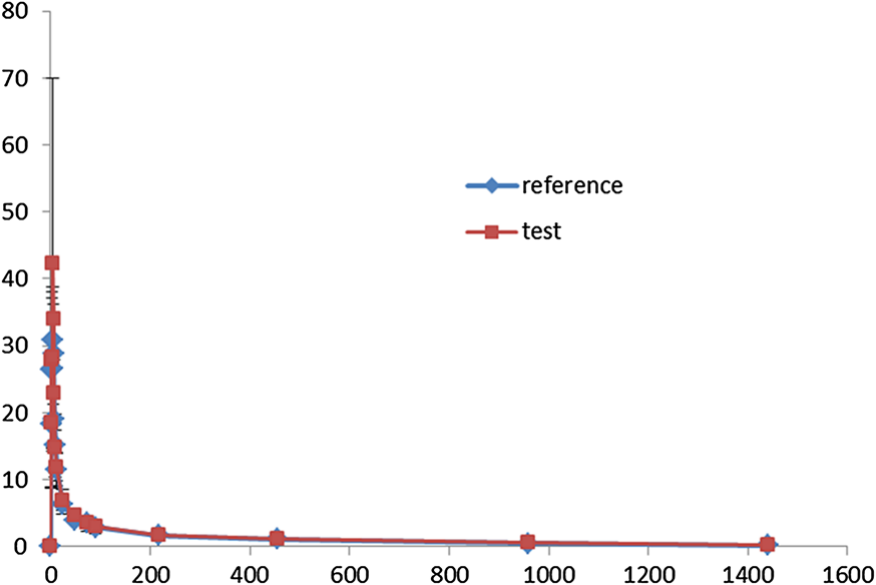
The mean (SD) concentration-time curves of HCQ (0–60d) after administration of the test formulation (Jiangsu Shenhua Pharmaceutical Co., Ltd., Jiangsu, China) and reference formulation (Shanghai Zhongxi Pharmaceutical Co., Ltd., Shanghai, China) in 20 healthy Chinese male volunteers

The mean ± SD main pharmacokinetic parameters *C*_max_ (index of the rate of absorption), AUC0–60d, and AUC_0−∝_ of the test and reference formulations (*n* = 20) are shown in Table 4. The mean (SD) concentration-time curves of HCQ after administration of the two formulations are shown in the Fig. 3. The 90% CIs of the ratios (test:reference) for the log-transformed *C*_max_ and AUC0-60d were 103.8–142.3% and 100.0–114.2%, respectively. The relative bioavailability was 109.5% (according to AUC_0–60d_) and 110.7% (according to AUC_0–∝_).

**Table 4:** Mean ± SD main pharmacokinetic parameters of the test and reference formulations (*n* = 20)

Pharmacokinetic parameters	Reference formulation (mean ± SD)	Test formulation (mean ± SD)	90% Confidence limit (%)
*T*_max_ (h)	3.65 ± 1.14	3.85 ± 1.04	
*C*_max_ (ng/ml)	34.3 ± 9.5	44.1 ± 27.6	103.8–142.3
AUC_0–60d_ (ng h/ml)	1679 ± 385	1789 ± 383	100–114.2
AUC_0–∞_ (ng h/ml)	1819 ± 417	1950 ± 435	100–115.5
*t*_1/2_ (h)	272 ± 76	298 ± 105	

Reference formulation: Shanghai Zhongxi Pharmaceutical Co., Ltd., Shanghai, China

Test formulation: Jiangsu Shenhua Pharmaceutical Co., Ltd., Jiangsu, China

#### 0–72h

**Fig. 4:**
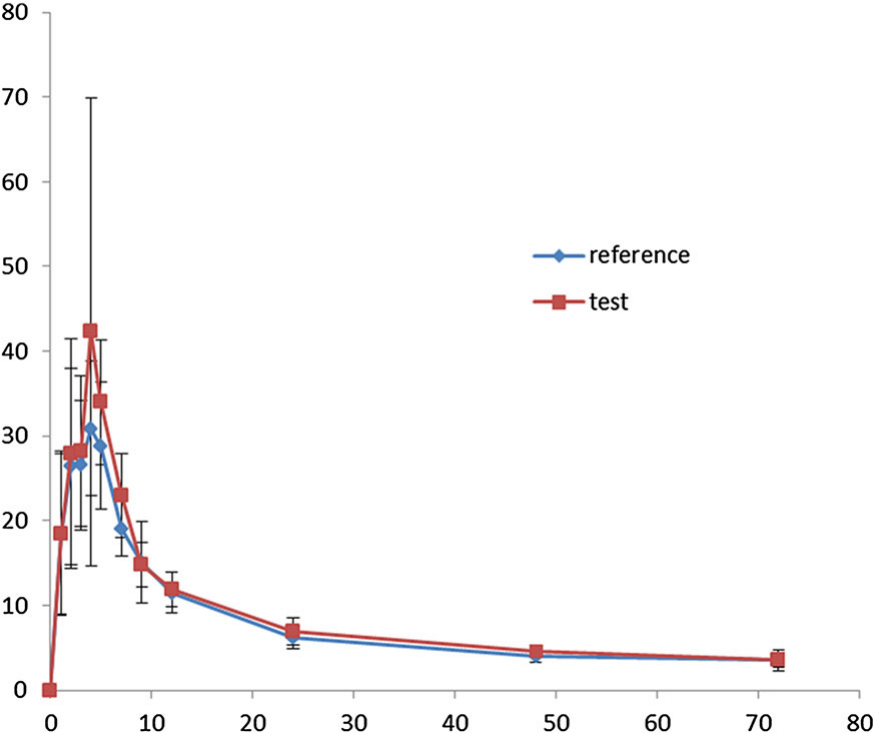
The mean (SD) concentration-time curves of HCQ (0–72h) after administration of the test formulation (Jiangsu Shenhua Pharmaceutical Co., Ltd., Jiangsu, China) and reference formulation (Shanghai Zhongxi Pharmaceutical Co., Ltd., Shanghai, China) in 20 healthy Chinese male volunteers

The mean ± SD main pharmacokinetic parameters *C*_max_ (index of the rate of absorption), AUC0–72h, and AUC_0–∝_ of the test and reference formulations (*n* = 20) are shown in Table 5. The mean (SD) concentration-time curves of HCQ after administration of the two formulations are shown in the Fig. 4. The 90% CIs of the ratios (test:reference) for the log-transformed *C*_max_ and AUC0–72h were 103.8–142.3% and 104.8–117.2%, respectively. The relative bioavailability was 111.8% (according to AUC_0–72h_) and 105.9% (according to AUC_0–∝_).

**Table 5:** Mean ± SD main pharmacokinetic parameters of the test and reference formulations (*n* = 20)

Pharmacokinetic parameters	Reference formulation (mean ± SD)	Test formulation (mean ± SD)	90% Confidence limit (%)
*T*_max_ (h)	3.65 ± 1.14	3.85 ± 1.04	
*C*_max_ (ng/ml)	34.3 ± 9.5	44.1 ± 27.6	103.8–142.3
AUC_0–72h_ (ng h/ml)	559 ± 94.1	617 ± 88.9	104.8–117.2
AUC_0–∝_ (ng h/ml)	753 ± 251	749 ± 108	92.9–112.6
*t*_1/2_ (h)	34.0 ± 16.9	32.3 ± 14.1	

Reference formulation: Shanghai Zhongxi Pharmaceutical Co., Ltd., Shanghai, China

Test formulation: Jiangsu Shenhua Pharmaceutical Co., Ltd., Jiangsu, China

Both the mean values and standard deviations of the main pharmacokinetic parameters such as *C*_max_, *T*_max_, AUC_0–60d_, and AUC_0–∝_ were found to be close between the test and reference preparations. In addition, the calculated 90% confidence interval for mean *C*_max_, AUC_0–60d_, and AUC_0–∝_ of the two drugs lay within the SFDA’s accepted range of 80–125%. Therefore, it could be concluded that the two hydroxychloroquine preparations analyzed were bioequivalent in terms of the rate and extent of absorption.

### Tolerability

No serious or unexpected adverse events were observed.

## Discussion

The aim of this study was to compare the bioavailability of the test formulation with that of the reference formulation, intending to acquire regulatory approval for the test formulation of HCQ. In this study, the AUC_0–*t*_, AUC_0–∝_, and *C*_max_ of HCQ were defined as the main parameters in order to assess the bioequivalence between both preparations. The criteria according to the SFDA’s guidelines for bioequivalence are the 90% CIs of the test/reference geometric means ratio in the range of 80–125% for the AUC and 70–143% for *C*_max_ [15]. The ANOVA results of this study showed that the formulation, period, and sequence had no statistically significant effect on the AUC_0–*t*_, AUC_0–∝_, and *C*_max_ of HCQ. Chinese regulatory authorities do not require the testing of food effects in relative bioavailability studies. Therefore, we only conducted this study under the fasting condition.

In the bioequivalence study of HCQ sulfate tablets (0–60 days), the ANOVA analysis results suggested that the main pharmacokinetic parameters are in accordance with the pharmacokinetics characteristic of a long half-life drug. As previously mentioned, the intra-subject bioavailability of HCQ is consistent. Therefore, according to the guideline of the FDA, for drugs that demonstrate low intra-subject variability in distribution and clearance, an AUC truncated at 72 h (AUC0–72h) can be used in place of an AUC_0–*t*_ or AUC_0–∝_ [17]. This sample collection time was adequate to ensure completion of gastrointestinal transit (~2–3 days) of the drug product and absorption of the drug substance. Therefore, this study also evaluated the bioequivalence of HCQ between a test preparation and reference preparation in 0–72 h (AUC0–72h); the results showed that both formulations are bioequivalent. The results from the two studies let us confirm that when comparing the two formulations’ bioequivalence for this kind of long half-life of drugs such as the HCQ tablets, a bioequivalence of 0–72 h is available to evaluate the bioavailability.

There are some potential limitations to the study. It was an open-label study, so it might not have objectively addressed the safety profiles of the test formulation. Moreover, the data of this study were obtained from healthy Chinese males under fasting conditions; thus, the findings cannot be expanded to predict therapeutic equivalence in patients in clinical practice.

## Conclusion

Based on the pharmacokinetics and the results of this study, it was concluded that the test and reference formulations of HCQ met the Chinese criteria for assuming bioequivalence. Both formulations were well tolerated in the population studies. Moreover, the results of applying a truncated AUC method in this study showed that this method is acceptable for estimating the relative bioavailability of HCQ.
